# Differential between-therapist effects in more versus less standardized therapies for depression

**DOI:** 10.1080/28324765.2024.2379248

**Published:** 2024-07-23

**Authors:** Anuj H. P. Mehta, Michael J. Constantino, Jack J. M. Dekker, Alice E. Coyne, Averi N. Gaines, Henricus L. Van, Jaap Peen, Frank J. Don, Ellen Driessen

**Affiliations:** a Department of Psychological and Brain Sciences, University of Massachusetts Amherst, Amherst, MA, USA; b Faculty of Behavioural and Movement Sciences, Vrije Universiteit Amsterdam, Amsterdam, The Netherlands; c Arkin Mental Health Care, Amsterdam, The Netherlands; d Department of Psychology, American University, Washington, DC, USA; e Depression Expertise Center, Pro Persona Mental Health Care, Nijmegen, The Netherlands; f Department of Clinical Psychology, Behavioural Science Institute, Radboud University, Nijmegen, The Netherlands

**Keywords:** therapist effects, treatment standardization, cognitive behavioral therapy, psychodynamic therapy, depression

## Abstract

Psychotherapists can differ in their effectiveness. Yet, more research is needed to determine the generalizability of between-therapist effects to cultures beyond the US and UK, and whether these effects differ by treatment context. Addressing these gaps, we examined therapist effects in a randomized trial comparing cognitive behavioral therapy (CBT) versus psychodynamic therapy (PDT) for depression in the Netherlands, hypothesizing that therapists would explain significant outcome variance across both treatments. We also explored whether the size of therapist effects differed by degree of treatment standardization; in this trial, CBT was structured and manualized, whereas PDT was flexible and principle driven. Patients were 254 adults who received 16 sessions of CBT or PDT from 59 therapists nested within the condition. As predicted, multilevel models revealed significant therapist effects (explaining 3–8% of variance) on patient depression and general distress outcomes. Moreover, PDT therapists (10–16%) accounted for more outcome variance than CBT therapists (2–6%), though differences were only statistically significant for the clinician-rated depression outcome. Results extend the cultural “reach” of therapist effects. Moreover, they highlight treatment standardization as one possible determinant of effectiveness differences, which necessitates determining for which therapists such standardization is most important for optimizing their patient’s outcomes.

It is well established that patients vary considerably in their psychotherapy outcomes (Lutz et al., 2021). Although such variability is often primarily attributed to differences between patients (see Constantino, Boswell, & Coyne, 2021) or treatment modalities (see Barkham & Lambert, 2021), outcomes can also be influenced by the treating therapist. Indeed, ample research has revealed general between-therapist effectiveness differences across diverse treatment settings (e.g., clinical trials, naturalistic care), treatment approaches (e.g., integrative, cognitive behavioral), and patient presenting concerns (e.g., depression, quality of life deficits), including when controlling for various patient case-mix variables (Coyne, 2024; Nissen-Lie et al., 2024; Wampold & Owen, 2021). These therapist effects are also clinically meaningful, explaining an average of approximately 5% of patient outcome variance (Baldwin & Imel, 2013; Johns et al., 2019). As a more concrete example of clinical impact, one study demonstrated that patient recovery rates for above-average therapists were almost twice the size of below-average therapists (i.e., 77% vs. 46%, respectively; Saxon & Barkham, 2012).

Notably, most research on between-therapist effects has been conducted in the United States of America (US) or United Kingdom (UK). For example, in the previously cited Johns et al. (2019) systematic review of 20 studies, 10 included a US-based sample and seven included a UK-based sample. Among the remaining three studies, one had a mix of German, British, and US participants (i.e., Schiefele et al., 2017). A second study had participants from Sweden and the US (i.e., Nissen-Lie et al., 2016). Finally, the third study, which was the only one without any participants from the US or UK, was conducted in the Netherlands (Wiborg et al., 2012). In this latter study of cognitive behavioral therapy (CBT) for chronic fatigue syndrome, the therapist explained 21% of patient outcome variance. Similarly, in the previously referenced Baldwin and Imel (2013) meta-analysis, only one of the 46 included studies was conducted in the Netherlands (van Minnen et al., 2003). In this study of behavioral therapy (BT) for trichotillomania, the therapist effect ranged from 0 to 35%.

Although these results outside of the US and UK samples preliminarily extended the generalizability, or cultural “reach”, of therapist effectiveness differences, more research of this type is needed, including by replicating the existence and size of the therapist effect in Dutch samples receiving other types of treatments for other presenting concerns. Although psychotherapies may look largely similar across Western, industrialized countries, subtle differences in training and clinical practices may exist that could influence the generalizability of results between such regions. And if differences in the size of between-therapist effects existed, it would necessitate more *culturally contextualized* research on unpacking and clinically leveraging therapist effectiveness differences. However, if there were no seeming cultural differences (i.e., the percentage of variance explained was largely comparable), it may point to there being more generally applicable determinants of therapist effects and principles for harnessing them.

Current limitations on cultural reach notwithstanding, it is also important to note there is heterogeneity in the size of therapist effects. An early meta-analysis demonstrated that the amount of patient outcome variance explained by the therapist ranged from 0 to 48% (Crits-Christoph et al., 1991). Similarly, a subsequent meta-analysis revealed a range of 0 to 55% (Baldwin & Imel, 2013). At present, variables that explain such effect size differences are mostly patient and treatment factors. For example, the influence of the provider on outcome is greater for, respectively, patients with higher versus lower presenting symptom severity (Johns et al., 2019) and treatments conducted in naturalistic versus controlled settings (Baldwin & Imel, 2013; Johns et al., 2019). Although the precise reasons for these moderator findings are unknown, researchers have offered several speculations.

For instance, it is possible that therapist effects are less pronounced in controlled clinical trials because the patients in them are often homogenous (e.g., those with a complex symptom presentation may be excluded) and their treating therapists are intentionally (vs. randomly) selected because of their putative expertise (Wampold & Owen, 2021). Additionally, or alternatively, it is plausible that therapist effects may be attenuated when therapists administer a specific bona fide treatment in a more standardized and manualized manner (which is often a central feature of clinical trials) and exaggerated when therapists administer the same, or a different, bona fide treatment in a less structured and more flexible, principle-driven manner (which is often a central feature of routine care; Crits-Christoph et al., 1991). However, intentionally testing this latter speculation in a controlled, comparative trial is challenging given that intervention studies are rarely designed to directly compare a more manualized bona fide treatment with a distinct and more principle-driven bona fide treatment.

Fortunately, one randomized controlled trial (RCT; Driessen et al., 2013) embodied this rare design feature by comparing two empirically supported treatments for Dutch adults with major depression—a highly structured and manualized CBT to a more flexible and principle-driven psychodynamic therapy (PDT). Drawing on these data, the present study first aimed to replicate the general presence of therapist effects on patient outcomes in a rather novel cultural context. Based on the broader extant literature and the two prior studies on therapist effects solely with Dutch patients (van Minnen et al., 2003; Wiborg et al., 2012), we hypothesized there would be significant therapist caseload-level effectiveness differences across both treatments. Second, we examined whether the size of such effects differed by treatment type (viz. degree of standardization). Given the lack of prior direct research on this question, this second aim was exploratory. Again, though, one might speculate conceptually that therapist differences may be attenuated in a more standardized therapy, such as CBT in this trial, and exaggerated in a more flexible and principle-driven therapy, such as PDT in this trial.

## Method

### Dataset overview

Data for this novel secondary analysis derived from the aforementioned RCT that compared the efficacy of CBT and PDT for adults with major depression (Driessen et al., 2007, 2013). Both treatments were administered individually and in person across 16 outpatient sessions over 22 weeks. Briefly, both treatments demonstrated comparable depression remission rates and clinician- and patient-rated symptom levels at posttreatment and 1-year follow-up. Given our focus on therapist caseload-level effectiveness, we included in the present study only therapists who had treated at least two patients (as such nesting is a requirement for therapist-level analysis). Moreover, given our interest in examining treatment condition as a moderator of therapist effectiveness differences, it was important we focus solely on therapists who administered only one of CBT or PDT. As a small number of therapists were crossed over condition, several other exclusions were necessary. Finally, from the remaining therapist subsample, we then excluded patients who did not provide any outcomes data beyond baseline. These therapist- and patient-level exclusions are described more fully in the next section.

### Participants

#### Patients

In the original Driessen et al. (2013) trial, consenting patients were 341 (*n*s = 164 & 177 in CBT & PDT, respectively) adults (aged 18–65) who met criteria for a major depressive episode, as per the Diagnostic and Statistical Manual of Mental Disorders 4th ed. (*DSM–IV*; American Psychiatric Association, 1994), and had a Hamilton Depression Rating Scale (HAM-D; Hamilton, 1960) score ≥ 14. Exclusion criteria were: (a) meeting criteria for psychotic symptoms or bipolar disorder, (b) severe suicidality that warranted a more intensive level of care than outpatient therapy, (c) substance misuse in the past 6 months, (d) pregnancy, (e) an inability to complete the trial’s protocol, and (f) use of medications that might influence one’s current psychological functioning. If a patient and the intake psychiatrist deemed an antidepressant medication to be ineffective, that individual was still eligible for the study after a washout period. As noted, for the present study, some patients were excluded if their therapist treated only them (which precluded therapist-level analysis) and/or they did not provide any outcomes data beyond baseline, which resulted in a subsample of 254 patients (*n*s = 132 & 122 in CBT & PDT, respectively). Table 1 displays the subsample’s descriptive statistics for their demographic characteristics, as well as their baseline depression and general distress severity (as per the three outcome measures described below). The treatment conditions differed only on one demographic characteristic; subsample patients in CBT had a higher gross monthly income than in PDT (φ = .20, *p* = .039). Moreover, subsample patients differed from full-sample patients on only one variable; included patients were slightly older (*M*_age_ = 39.62; *SD* = 10.14) than excluded patients (*M*_age_ = 36.83; *SD* = 10.14; *t*(339) = −2.19, *p* = .029).

**Table 1.** Subsample patient baseline demographic characteristics and symptom severity by treatment condition (*N* = 254)

	CBT (*n* = 132)				PDT (*n* = 122)			
	*n*	%	*M*	*SD*	*n*	%	*M*	*SD*
*Demographic*								
Age (years)			38.94	9.84			40.35	10.44
Sex								
Male	40	30.30			40	32.79		
Female	92	69.70			82	67.21		
Cultural background								
Northwest European	78	59.09			65	53.28		
Other	54	40.91			57	46.72		
Marital Status								
Married	41	31.06			22	18.03		
Divorced	29	21.97			23	18.85		
Widowed	3	2.27			5	4.10		
Never married	58	43.94			71	58.20		
Other	1	0.76			1	0.82		
Living situation								
Living with at least one other person	87	65.91			74	60.66		
Living alone	44	33.33			40	32.79		
Other	1	0.76			7	5.74		
Missing	0	0.00			1	0.82		
Job status								
Currently working	51	38.64			47	38.52		
Receiving sickness benefits	28	21.21			17	13.93		
Receiving social security benefits	24	18.18			28	22.95		
Disabled	12	9.09			14	11.48		
Student	3	2.27			5	4.10		
Other	13	9.85			10	8.20		
Missing	1	0.76			1	0.82		
Education level								
Low	27	20.45			22	18.03		
Middle	56	42.42			58	47.54		
High	46	34.85			35	28.69		
Other	2	1.52			5	4.10		
Missing	1	0.76			2	1.64		
Main income before taxes*								
≤ €1,273 a month	38	28.79			46	37.70		
> €1,274 a month	75	56.82			53	43.44		
Missing	19	14.39			23	18.85		
*Symptom severity*								
Baseline HAM-D			23.49	5.14			23.58	5.51
Baseline IDS			43.26	10.19			42.76	12.28
Baseline OQ-45			92.82	22.97			91.54	22.18

CBT = Cognitive-behavioral therapy; PDT = Psychodynamic therapy; HAM-D = Hamilton Depression Rating Scale; IDS = Inventory of Depressive Symptomatology; OQ-45 = Outcome Questionnaire. Some *n*s total to less than the respective condition sample size due to missing data.

*In our subsample, these income levels differed significantly by treatment condition.

#### Therapists

In the original Driessen et al. (2013) trial, therapists were 93 (*n*s = 37 & 56 in CBT & PDT, respectively) psychologists or psychiatrists with at least a master’s degree. As noted, we excluded therapists who treated only a single patient. Moreover, there were 10 therapists who treated patients in both conditions. In half of these cases, the therapist saw multiple patients in one condition and only one patient in the other condition. In these instances, we excluded the single patient from the second condition in our analyses (as reflected in the previously stated patient subsample *n*). For the other five therapists, they saw only one patient in each condition. Thus, consistent with our previously noted therapist selection criteria, these five therapists and their patients were excluded (again, the patient exclusions were accounted for in the previously stated patient subsample *n*). After exclusions, we analyzed a subsample of 59 therapists (*n*s = 24 & 35 in CBT & PDT, respectively). Within this subsample, therapists primarily identified as female (62.7%), and their mean age was 41.27 years (*SD* = 9.68). In terms of clinical experience, they averaged 8.42 years (*SD* = 7.46). Most subsample therapists were masters or doctoral-level psychologists (61.0%); the remaining therapists were licensed psychiatrists (22.0%) or psychiatry residents (16.9%). Subsample therapists did not differ from full-sample therapists on any of these variables. However, among subsample therapists, the treatments differed on two of these variables. First, a greater percentage of therapists in the PDT condition identified as male (*n* = 18; 51.4%) than in the CBT condition (*n* = 4; 16.7%), χ^2^ (1) = 7.36, *p* = .007, φ =.35. Second, the treatments differed in the number of psychologists and psychiatrists. In CBT, there were no psychiatrists (i.e., all 24 therapists were psychologists). In PDT, there were 23 psychiatrists/psychiatry residents and 12 psychologists (χ^2^ (1) = 25.85, *p* < .001; φ = 0.66).^1^ Finally, each subsample therapist treated an average of 4.31 patients (*SD* = 2.68; range = 2–15).

### Treatments

Both treatments were provided weekly for the first 10 weeks and biweekly over the remaining 12 weeks. Moreover, therapists in both CBT (Molenaar et al., 2009) and PDT (de Jonghe, 2005) delivered treatment according to their respective published manual (see Driessen et al., 2013). Briefly, CBT strategies centered on cognitive restructuring and behavioral activation, using between-session homework to reinforce such work. As noted previously, the CBT condition was highly structured in its session-by-session content and process. PDT strategies centered on exploring, emotionally experiencing, and resolving the interpersonal patterns that underlie one’s depression. As noted, the PDT condition was less structured than CBT; rather, it was more flexibly principle-driven in its content and process. In terms of training, all subsample therapists first completed an intensive, multiday course in either CBT or PDT. Second, therapists treated a pilot case, for which they received feedback from expert trial supervisors. Finally, all therapists treated trial cases with ongoing peer supervision (see Driessen et al., 2013, for additional details). As an additional design feature of the Driessen et al. (2013) trial, for ethical purposes, patients (*n* = 129) with severe depression at baseline (HAM-D > 24) and those (*n* = 21) who developed severe symptoms during the trial were offered pharmacotherapy in addition to CBT or PDT. Of these patients, 142 took the recommended medication.^2^ As discussed in the subsequent data analysis section, we included this adjunctive treatment variable as a covariate in our analyses.

### Measures

The present secondary analyses focused on the treatment outcomes of independent clinician- and patient-rated depression and patient-rated general psychological distress. Clinician-rated depression was assessed with the widely used HAM-D (Hamilton, 1960). Completed by trained master’s-level trainees in clinical psychology, the HAM-D consists of 17 items (rated on a 0–2 or 0–4 scale) that assess depression-specific symptoms, such as anhedonia, sleep and appetite problems, suicidal ideation, and feelings of guilt. Higher total scores on the HAM-D, which we used in the present study, reflect more severe depression (possible range = 0–52). The psychometric properties of the HAM-D have been well-established (Bagby et al., 2004). In the Driessen et al. (2013) trial, interrater reliability on this measure was high (.97), and internal consistency was good across all study-relevant time points (average *α* = .74).

Patient-rated depression was assessed with the Inventory of Depressive Symptomatology Self-Report (IDS-SR; Rush et al., 1986). The IDS-SR includes 28 items (rated from 0–3) that assess five dimensions: vegetative symptoms, cognitive changes, mood disturbance, endogenous symptoms, and anxiety symptoms. Higher total scores on the IDS-SR, which we used in the present study, reflect more severe depression (possible range = 0–84). The psychometric properties of IDS-SR have been well-established (Rush et al., 1996; Trivedi et al., 2004). In the present study, this measure demonstrated good internal consistency across all study-relevant time points (average *α* = .83).

To assess general psychological distress, patients completed the 45-item Outcome Questionnaire (OQ-45; de Beurs et al., 2005; Lambert et al., 1996). The OQ-45 items are rated from 0–4, with higher total scores (which we used in the present study) reflecting more severe general distress (possible range = 0–180). The OQ-45’s psychometric properties are also well-established (de Beurs et al., 2005; Lambert et al., 1996). In the present study, this measure demonstrated excellent internal consistency across all study-relevant time points (average *α* = .93).

### Procedure

See Driessen et al. (2013) for full details regarding participant flow through the trial. Most relevant to the present study, patients meeting inclusion criteria for a major depressive episode based on the Mini-International Neuropsychiatric Interview–Plus (Sheehan et al., 1998) and who gave written informed consent to participate in the trial were randomly assigned to CBT or PDT, which was administered at one of the three psychiatric outpatient clinics in Amsterdam. The HAM-D was assessed at baseline, week 5, week 10, and posttreatment. The IDS-SR and OQ-45 were assessed at baseline, week 10, and posttreatment. The institutional review board of the Dutch Union of Medical Ethics Trial Committees approved both the parent trial and subsequent analyses of deidentified data (Driessen et al., 2007, 2013). The present secondary analyses were preregistered through OSF (https://osf.io/u385z/?view_only=822049570ea448cc91f9e7cc6339e874).

### Data analyses

We first calculated descriptive statistics for all study variables in our patient and therapist subsamples to inform whether we needed to conduct any transformations (due to non-normal distributions) and/or sensitivity analyses (due to the presence of outliers with values greater than ±3 standard deviations from the mean). Next, we conducted all primary analyses using multilevel structural equation modeling (MSEM; Preacher et al., 2016), as implemented in the Mplus program (Muthén & Muthén, 2017). This modeling approach allowed us to account for the nested data structure to appropriately estimate therapist effects. Across all models, because variance components (a key focus of this study) are typically not normally distributed, we used the Bayesian estimator to generate 95% credible intervals (CIs) that do not assume normality. Using this approach, 95% CIs that do not include zero are considered statistically significant (B. Muthén & Asparouhov, 2012). We used non-informative priors to allow the models to be estimated based solely on the data. This approach uses the Bayesian corollary of full information maximum likelihood estimation to address missing data (B. Muthén & Asparouhov, 2012). Thus, as noted, we retained all participants in our patient and therapist subsamples who completed at least one assessment of any study outcome variable that was included in a particular model, which resulted in a modified intent-to-treat approach. Because the amount of missing data differed by outcome variable, the number of patients and therapists included in the analyses varied. Specifically, all 254 subsample patients and 59 therapists were included in the models examining clinician-rated depression; 193 patients and 53 were included in the models examining self-reported depression; and 169 patients and 45 therapists were included in the models examining patient-rated general psychological distress.

To test both of our aims of examining between-therapist differences in primary and secondary patient treatment outcomes, we fit 3-level models with within-patient change over time at level 1, between-patient (within-therapist) differences at level 2, and between-therapist differences at level 3. We fit one model for each outcome and, from each, we calculated an intraclass correlation (ICC; Raudenbush & Bryk, 2002), which quantified the percentage of total outcome variance across therapy explained by the therapist (i.e., the therapist effect). Across all models, we included adjunctive pharmacotherapy status and the relevant baseline severity variable as covariates.

Given our interest in examining the amount of variance explained by the therapist within each treatment condition (aim 2), we fit dual intercept models; that is, we dropped the overall (default) model intercept and instead included two indicator variables, one for CBT (CBT = 1; PDT = 0) and one for PDT (PDT = 1; CBT = 0), at level 1. This created two separate random intercepts, which enabled separate estimation of patient- and therapist-level variability by treatment condition. For aim 1, we constrained (fixed) the patient- and therapist-level variability (at levels 2 and 3, respectively) in the random intercepts to be equal across conditions. This allowed us to calculate an overall ICC for each outcome variable.

Next, for aim 2, we freed the patient- and therapist-level variance components, which allowed us to test whether the amount of therapist-level variability significantly differed by treatment condition.^3^ Specifically, we used the “model constraint” function within Mplus to compute the treatment-specific ICC for each condition (i.e., amount of variance explained by the therapist within CBT and PDT) and test whether they significantly differed using a z-test (the default in Mplus). The full Mplus syntax and multilevel equation for our models are included in the online supplement.

Although the analyses for this study were preregistered via OSF, we deviated from our original analytic plan in two ways. First, as noted in a formal update to our preregistered analytic plan, we became aware of a mistake in the language of our preregistration. Specifically, it was never our intention to look at treatment condition as a *predictor* of therapist-level outcome; instead, consistent with our preregistered aims, our intention was to examine whether the amount of variability attributed to the therapist differed by treatment condition. We updated our analytic plan accordingly. Second, in response to feedback during the peer review process, we updated our analytic approach for examining differences in therapist effects between the two treatment conditions. Namely, we decided to use the dual intercept approach described above to allow us to directly and inferentially (rather than descriptively) test for differences in ICCs between the two treatment conditions. When conducting these new analyses, we noticed potentially different effects of medication status on patient outcomes by treatment condition; therefore, to more conservatively estimate any differences in therapist effects by treatment condition, we included all covariates in our primary models. Importantly, we consider these to be relatively minor changes to our analytic approach, and we did not change our preregistered research aims/questions or measured variables.

## Results

The three outcome variables were acceptably normally distributed, and we did not identify any significant outliers. For aim 1, for the first outcome of clinician-rated depression (i.e., HAM-D), therapists accounted for a statistically significant 6% of the variance (*τ*_β00_ = 3.30, *p* < .001; 95% CI 0.93, 8.89). For the second outcome of patient-rated depression (i.e., IDS-SR), therapists again accounted for a statistically significant 8% of the variance (*τ*_β00_ = 15.57, *p* < .001; 95% CI 0.96, 39.02). For the third outcome of patient-rated general psychological distress (i.e., OQ-45), therapists accounted for a statistically significant 3% of the variance (*τ*_β00_ = 26.18, *p* < .001; 95% CI 2.84, 142.33). For the full results of these models, see Supplemental Table 1.

For aim 2, for the first outcome of clinician-rated depression, therapists accounted for a statistically significant 16% of the variance in PDT (*τ*_β00_ = 8.75, *p* < .001; 95% CI 3.37, 20.46) and a statistically significant 2% of the variance in CBT (*τ*_β00_ = 1.04, *p* < .001; 95% CI 0.13, 4.97). Moreover, this difference in therapist-level variability across conditions was statistically significant (difference in ICC = 0.14, *p* = .004, 95% CI 0.03, 0.29). For the second outcome of patient-rated depression, therapists accounted for a statistically significant 16% of the variance in PDT (*τ*_β00_ = 28.76, *p* < .001; 95% CI 4.72, 74.17) and a statistically significant 6% of the variance in CBT (*τ*_β00_ = 13.40, *p* < .001; 95% CI 1.81, 71.15). However, the difference in therapist-level variability between conditions was not statistically significant (difference in ICC = 0.08, *p* = .22, 95% CI −0.16, 0.28). For the global distress outcome, therapists accounted for a statistically significant 10% of the variance in PDT (*τ*_β00_ = 66.59, *p* < .001; 95% CI = 8.92, 286.54), and a statistically significant 4% of the variance in CBT (*τ*_β00_ = 37.66, *p* < .001; 95% CI 1.51, 211.70). However, this difference in therapist-level variability between conditions was not statistically significant (difference in ICC = 0.04, *p* = .27, 95% CI −0.12, 0.26). For the full results of these models, see Supplemental Table 2.

## Discussion

Drawing on data from an RCT that compared the efficacy of a structured and manualized CBT to a more flexible and principle-driven PDT in the treatment of Dutch adults with major depression (Driessen et al., 2013), we examined the amount of outcome variance explained by the therapist and whether the size of this between-therapist effect differed by treatment condition. As hypothesized, and consistent with US- and UK-based samples (within which therapist effects have been most frequently studied), significant therapist effects emerged in the present sample—whether patient outcomes were clinician-rated or self-reported. Additionally, PDT therapists accounted for more outcome variance than CBT therapists across all outcomes, though these differences were only statistically significant for clinician-rated depression.

Our predicted aim 1 results extend the generalizability of therapist effectiveness differences to a Dutch sample receiving either CBT or PDT for major depression. Thus, any subtle differences in the populations receiving psychosocial services that might exist between the Netherlands and US or UK treatment contexts did not change the overall presence of a significant between-therapist effect across two empirically supported treatments for depression. Moreover, the size of this effect in the present study (ranging from 3–8% of variance explained across different outcome variables) fits a robust pattern in the broader literature; namely, the range is consistent with data from other RCTs, for which the therapist tends to explain less outcome variance (~3%, on average) than in naturalistic treatment settings (~7%, on average; see Baldwin & Imel, 2013).

Notably, though, the amount of outcome variance explained by the therapist in the present study was generally lower than it was in the other RCT with a fully Dutch sample (i.e., up to 35% in the van Minnen et al., 2003, study of BT for trichotillomania, as estimated in; Baldwin & Imel, 2013). It is plausible that this discrepancy is a function of the van Minnen et al. (2003) trial having had only five therapists treating an average of 2.50 patients; that is, such low power may have limited the reliability of therapist effectiveness differences beyond sampling error (Baldwin & Imel, 2013). Alternatively, it is possible the influence of the therapist on patient outcomes truly differs by patients’ presenting problem; in this case, the therapist may have a bigger or more variable impact when treating a rarer and arguably more recalcitrant problem like trichotillomania versus depression. Future research is needed with well-powered studies to better explicate the existence and size of therapist effects in diverse cultural contexts (perhaps especially those more dissimilar to the US, UK, and Netherlands) and for specific presenting concerns.

Such potential power limitations and nuances aside, a primary clinical implication of the mounting (and thus far cross-cultural) evidence of between-therapist effectiveness differences is that clinicians should consider measuring and monitoring their outcomes (Muir et al., 2019; Rousmaniere et al., 2020). Even in the context of tightly controlled trials with standardized training and intensive supervision, therapists are not equally effective. With a growing personal database, therapists may become more accurate in determining their overall effectiveness (see Constantino et al., 2023). Moreover, when treating different types of patients and/or using multidimensional outcome tools, therapists can begin to learn the patients for whom they are currently more or less able to help (see Coyne, 2024). Such therapist-level feedback can be used to guide therapist strengths-based specialization or to personalize training and continuing education practices to improve areas of weakness (Constantino, in press). Additionally, at a system’s level, therapist data can be used to intentionally assign patients to the therapists most likely to help them at that cross-section in time (Constantino, Boswell,Coyne, Swales et al., 2021; Delgadillo et al., 2020).

Our exploratory aim 2 results revealed that the degree of treatment standardization within an RCT for depression *may* be a potential determinant of between-therapist effectiveness differences. Namely, at least in the context of this Dutch sample and for clinician-rated depression, therapists explained more outcome variance when they used flexible and principle-driven PDT compared to structured and manualized CBT to treat depression. Although this result needs to be interpreted cautiously given the lack of differences for the patient self-reported outcomes, it may again mirror the broader literature; therapists generally explain more outcome variance when practicing in naturalistic treatment settings—where they are largely unconstrained by treatment prescription and are therefore perhaps more flexible—relative to RCTs (Baldwin & Imel, 2013).

Clinically, this present result preliminarily indicates that administering a more versus less structured treatment protocol (in this case as cultivated by the research design) may attenuate the influence the provider has on their patients’ improvement—again, at least when outcome is assessed by a clinician observer. Translationally, though, the implication is complex. On the one hand, it is possible that for underperforming therapists, greater structure and standardization via treatment protocols may have the benefit of raising their effectiveness “floor”, perhaps because they become better at intentionally providing a uniformly compelling treatment rationale that increases patients’ expectation that the treatment will be helpful. Supporting this notion, research has demonstrated that both higher patient-perceived treatment credibility and higher outcome expectation have been consistently associated with better outcome (Constantino, Coyne, et al., 2018; Constantino, Vîslă, et al., 2018).

On the other hand, highly structured treatments may also have the unintended effect of lowering the effectiveness “ceiling” of the therapists who are highly successful without such structure and standardization, perhaps *because* of their natural flexibility and responsiveness. Supporting this notion, research has demonstrated that when a therapist uses clinical strategies more flexibly when treating a given patient, that unique dyad shows better therapy process (Goldman et al., 2013) and that individual patient demonstrates more improvement (Katz et al., 2019). Notably, such *adherence flexibility* has involved the use of techniques that might be considered irrelevant or even proscribed if the provider’s goal was to remain highly faithful to a manualized and sequenced set of theory-specific interventions.

Of course, the wider range of therapist effects in the present PDT condition also renders the clinical implications of therapist flexibility complex. Whereas it may allow some therapists to “shine”, it may render others ineffective, or even harmful. Thus, at the systems level, a care network may need to weigh a desire to have more consistently average-to-good outcomes across their providers (for which using more standardized treatments may help) or to tolerate some ineffectiveness among some providers to achieve some excellent patient outcomes. Future research will need to determine, taking multiple patient-, therapist-, and system-level determinants into account, how best to balance such decisions in a way that results in the greatest public health benefit.

For example, to the extent that treatment standardization has the potential to strengthen or dampen a therapist’s effectiveness, perhaps the key is to determine when standardization helps bring up the clinicians who most need it (raises their floor) versus holds back the ones who do well without it (lowers their ceiling). That is, future research can focus on learning the determinants of who is optimally effective under which standardization conditions. (To use a metaphor, we would want to learn what makes one comedian funny because of deliberately practicing their scripted routine and what makes another funny because of improvising without structure.) Returning to the therapy sphere, once we learn such determinants, we can also personalize therapist training. For example, we can learn who most needs intensive supervision or consultation to help provide the treatment roadmap or “script” versus who just needs a breadth of therapy knowledge with which to improvise (with little oversight or attempt to shape).

Several limitations characterized this study. First, as noted, our second aim was limited by low power at the therapist level—both in terms of the number of therapists and especially the number of patients they treated. More specifically, although we inferentially tested whether differences in ICCs between treatment conditions were statistically significant, these analyses were underpowered, which could be one explanation as to why the differences were only statistically significant for one outcome. Supporting this view, it is worth noting the analysis involving the clinician-rated depression outcome had the highest number of therapists and patients, which resulted in the highest power. Therefore, future research with larger therapist and patient-within-therapist samples is needed to more confidently establish the size (and generalizability across outcomes) of the therapist effect in Dutch samples; Schiefele et al. (2017) recommend at least 100 therapists seeing at least 10 patients each. Although these larger samples may be difficult to achieve in a single RCT, there may be promise in drawing on individual participant data meta-analyses to do so (e.g., Driessen et al., 2024).

Second, although the RCT’s unique design feature allowed us to ostensibly examine the degree of treatment standardization as a potential determinant of the size of the between-therapist effect, it remains possible that the differences observed had more to do with the interventions used in CBT versus PDT (perhaps even if they were *equally* structured and manualized). For example, the very nature of PDT (with its focus on the unique therapy relationship as the primary mechanism of change) may generate more “room” for the therapist to variably influence patients’ outcomes than CBT (with its focus on teaching uniform strategies as the primary mechanism of change). Future research will need to determine if treatment standardization and type (at least regarding CBT and PDT) have unique influences on the size of the therapist effect or are essentially overlapping constructs.

Third, the RCT did not include a formal, observer-based assessment of therapist adherence to their respective treatment approach; thus, it is possible that CBT and PDT were less distinct on being structured and manualized versus flexible and principle-driven (which may have confounded the results). Fourth, as therapists were nested within treatment condition rather than being crossed over them, it is possible that any differences in the size of therapist effects between CBT and PDT were driven more by general therapist factors than the level of therapy standardization. In fact, one such factor that could have influenced the present results was therapist discipline type, which differed significantly between the treatment conditions. Although this variable did not predict general therapist-level outcome, we cannot rule out that it may have influenced the differences in ICCs between treatments. Finally, therapist race/ethnicity was not assessed in the Driessen et al. (2013) trial; thus, a key demographic of the present therapist sample was unknown.

Limitations notwithstanding, the findings extend the reach of the therapist effects literature to a Dutch sample with depression, and shed some preliminary light on one factor that may influence the size of such effects. With future research uncovering the skills or characteristics that differentiate the therapists who need structure versus those who thrive on flexibility, and any cultural differences in these determinants, the field can move toward more personalized and culturally contextualized therapist practices and trainings (Constantino, in press; Coyne et al., 2022).

## Supplementary Material

Supplement_FINAL.docx

## Data Availability

The data that support the findings of this study are not available publicly. However, data requests, including a detailed plan for how they will be used, should be directed via email to the last author, Dr. Ellen Driessen (ellen.driessen@ru.nl). Dr. Driessen reserves the right to deny individual share requests if the usage details are questionable or unclear.

## Notes

1. Importantly, none of the four therapist variables discussed in this section significantly predicted therapist effects on any of the patients’ treatment outcomes.

2. For our present patient subsample, this number was 100.

3. Although we were only interested in therapist-level outcome variability, we also freed the patient-level outcome variance across conditions to avoid falsely attributing any between-patient differences in outcome variability to the therapist.
