# Correction

**DOI:** 10.1080/28324765.2025.2457877

**Published:** 2025-01-28

**Article title**: Differential between-therapist effects in more versus less standardized therapies for depression

**Authors**: Anuj H. P. Mehta, Michael J. Constantino, Jack J. M. Dekker, Alice E. Coyne, Averi N. Gaines, Henricus L. Van, Jaap Peen, Frank J. Don, and Ellen Driessen

**Journal**: Cogent Mental Health

**DOI**: 10.1080/28324765.2024.2379248

This article was originally published with an error in the text. To rectify this, the following additional content has now been included at the middle of the Procedure section.

Before correction:

***Procedure***

See Driessen et al. (2013) for full details regarding participant flow through the trial. Most relevant to the present study, patients meeting inclusion criteria for a major depressive episode based on the Mini-International Neuropsychiatric Interview–Plus (Sheehan et al., 1998) were randomly assigned to CBT or PDT, which was administered at one of the three psychiatric outpatient clinics in Amsterdam. The HAM-D was assessed at baseline, week 5, week 10, and posttreatment. The IDS-SR and OQ-45 were assessed at baseline, week 10, and posttreatment. The institutional review board of the Dutch Union of Medical Ethics Trial Committees approved both the parent trial and subsequent analyses of deidentified data (Driessen et al., 2007, 2013). The present secondary analyses were preregistered through OSF (https://osf.io/u385z/?view_only=822049570ea448cc91f9e7cc6339e874).

After correction:

***Procedure***

See Driessen et al. (2013) for full details regarding participant flow through the trial. Most relevant to the present study, patients meeting inclusion criteria for a major depressive episode based on the Mini-International Neuropsychiatric Interview–Plus (Sheehan et al., 1998) and who gave written informed consent to participate in the trial were randomly assigned to CBT or PDT, which was administered at one of the three psychiatric outpatient clinics in Amsterdam. The HAM-D was assessed at baseline, week 5, week 10, and posttreatment. The IDS-SR and OQ-45 were assessed at baseline, week 10, and posttreatment. The institutional review board of the Dutch Union of Medical Ethics Trial Committees approved both the parent trial and subsequent analyses of deidentified data (Driessen et al., 2007, 2013). The present secondary analyses were preregistered through OSF (https://osf.io/u385z/?view_only=822049570ea448cc91f9e7cc6339e874).

