# Supplementary material for: Differential between-therapist effects in more versus less standardized therapies for depression
Source: Cogent Ment Health. 2024 Jul 23;3(1):2379248. doi: 10.1080/28324765.2024.2379248 (PMC12443010; doi:10.1080/28324765.2024.2379248)
Supplement: Supplement_FINAL.docx [file OAMH_A_2379248_SM0605.docx]

**Mplus Model Syntax**

**Aim 1 Syntax for Clinician-rated Depression**

TITLE: Aim 1 Therapist Effects on Clinician-Rated Depression;

DATA: FILE IS Data.dat;

DEFINE: CENTER hdrs1 (GROUPMEAN TID); !patient-level symptom severity covariate

VARIABLE: NAMES ARE PID TID Tx_cond Time HDRS Med_stat hdrs1 CBT SPSP;

USEVARIABLES ARE PID TID HDRS CBT SPSP Med_stat hdrs1;

CLUSTER IS TID PID;

MISSING ARE ALL (999);

WITHIN ARE CBT PDT HDRS;

BETWEEN ARE (PID) Med_stat hdrs1;

ANALYSIS: TYPE IS THREELEVEL RANDOM; ESTIMATOR=BAYES;

MODEL:

%WITHIN%

HDRS (w); !estimate within-patient variability in outcome

CBT_int | HDRS ON CBT; !create random intercept for CBT condition

PDT_int | HDRS ON PDT; !create random intercept for PDT condition

[HDRS@0]; !suppress overall model intercept

%BETWEEN PID%

CBT_int (bp); !fix patient-level variance to be equal across conditions

PDT_int (bp); !fix patient-level variance to be equal across conditions

CBT_int PDT_int ON med_stat (med); !control for covariates, fixed across conditions

CBT_int PDT_int ON hdrs1 (baseline); !control for covariates, fixed across conditions

SPSP_int WITH CBT_int@0;!fix correlation between intercepts to zero

%BETWEEN TID%

CBT_int (bt); !fix patient-level variance to be equal across conditions

PDT_int (pdt_bt); !fix patient-level variance to be equal across conditions

SPSP_int WITH CBT_int@0; !fix correlation between intercepts to zero

MODEL CONSTRAINT: NEW(ICC);

ICC = bt/(w+bp+bt); calculate overall ICC across both conditions

**Aim 2 Mplus Syntax for Clinician-rated Depression**

TITLE: Aim 2 Treatment Differences in Therapist Effects;

DATA: FILE IS Data.dat;

DEFINE: CENTER hdrs1 (GROUPMEAN TID); !patient-level symptom severity covariate

VARIABLE: NAMES ARE PID TID Tx_cond Time HDRS Med_stat hdrs1 CBT SPSP;

USEVARIABLES ARE PID TID HDRS CBT SPSP Med_stat hdrs1;

CLUSTER IS TID PID;

MISSING ARE ALL (999);

WITHIN ARE CBT PDT HDRS;

BETWEEN ARE (PID) Med_stat hdrs1;

ANALYSIS: TYPE IS THREELEVEL RANDOM; ESTIMATOR=BAYES;

MODEL:

%WITHIN%

HDRS (w); !estimate within-patient variability in outcome

CBT_int | HDRS ON CBT; !create random intercept for CBT condition

PDT_int | HDRS ON PDT; !create random intercept for PDT condition

[HDRS@0]; !suppress overall model intercept

%BETWEEN PID%

CBT_int (cbt_bp); !estimate between-patient variance in CBT condition

PDT_int (pdt_bp); !estimate between-patient variance in PDT condition

CBT_int PDT_int ON med_stat hdrs1; !control for covariates

SPSP_int WITH CBT_int@0;!fix correlation between intercepts to zero

%BETWEEN TID%

CBT_int (cbt_bt); !estimate between-therapist variance in CBT

PDT_int (pdt_bt); !estimate between-therapist variance in PDT

SPSP_int WITH CBT_int@0; !fix correlation between intercepts to zero

MODEL CONSTRAINT: NEW(cbt_icc pdt_icc diff_icc);

cbt_icc= cbt_bt/(cbt_bt+cbt_bp+w); !calculate CBT ICC

SPSP_icc= spsp_bt/(spsp_bt+spsp_bp+w); !calculate PDT ICC

diff_icc = SPSP_icc -cbt_icc; !test differences between ICCs

**General Multilevel Equation (Aim 2)**

**Level-1 Model**

Outcome*_ijk_* = *π_1jk_**CBT*_jk_* + *π_2jk_**PDT*_jk_* + *e_ijk_*

**Level-2 Model**

*π_1jk_* = *β_10k_* + *β_11k_**(Baseline Outcome*_jk_*) + *β_12k_**(Medication Status*_jk_*) + *r_1jk_*

*π_2jk_* = *β_20k_* + *β_21k_**(Baseline Outcome*_jk_*) + *β_22k_**(Medication Status*_jk_*) + *r_2jk_*

**Level-3 Model**

*β_10k_* = *γ_100_* + *u_10k_*

*β_11k_* = *γ_110_*

*β_12k_* = *γ_120_*

*β_20k_* = *γ_200_* + *u_20k_*

*β_21k_* = *γ_210_*

*β_22k_* = *γ_220_*

At level 1, outcome at time *i* for patient *j* treated by therapist *k* was predicted by a CBT indicator variable (CBT = 1; PDT = 0), a PDT indicator variable (PDT = 1; CBT = 0), and within-patient variance (*e_ijk_*). The inclusion of these two indicator variables resulted in separate intercepts (*π_1jk,_ π_2jk_*) for each treatment condition. At level 2, these treatment-specific intercepts dropped down to become the outcome variables and were predicted by the two covariates (patient-level differences in baseline outcome and medication status). Most relevant to the present study, a patient-level random effect was included for each of the intercepts (*r_1jk,_ r_2jk_*). These random effects represented the between-patient variability in outcome after removing the influence of medication status and baseline symptom severity. At level 3, a random effect was again included for each treatment-specific intercept (*u_10k,_ u_20k_*) to allow outcome to vary among therapists within each treatment condition. To ensure model identification, the two random intercepts were not allowed to correlate with one another (i.e., we fixed the covariance at levels 2 and 3 to 0). The variance explained by the therapist (i.e., the intraclass correlation [ICC]) was calculated separately for each treatment condition using the following formula: between-therapist variance/(within-patient variance + between-patient variance + between-therapist variance).

Although not of primary interest in this study, the fixed effects in the model represented: the average outcome level for patients in the CBT condition (*γ_100_*); the association between baseline outcome and subsequent outcome in the CBT condition (*γ_110_*); the association between medication status and outcome in the CBT condition (*γ_120_*); the average outcome level for patients in the PDT condition (*γ_200_*); the association between baseline outcome and subsequent outcome in the PDT condition (*γ_210_*); and the association between medication status and outcome in the PDT condition (*γ_220_*).

Supplemental Table 1

*Overall Therapist Effects on Patient Outcomes Across the CBT and PDT Conditions*

|  | Clinician-rated Depression | | Patient-rated Depression | | General Psychological Distress | |
| --- | --- | --- | --- | --- | --- | --- |
| **Fixed effects** | Coefficient | 95% CI | Coefficient | 95% CI | Coefficient | 95% CI |
| Outcome, *γ_000_* |  |  |  |  |  |  |
| Baseline outcome, γ_010_ | 0.77* | 0.47, 1.02 | 0.64* | 0.46, 0.85 | 0.24* | 0.02, 0.43 |
| Medication status, γ_020_ | 0.43 | -2.30, 3.90 | 3.93 | -1.16, 7.55 | 10.16* | 0.01, 1.35 |
| **Random effects** | Variance component | 95% CI | Variance component | 95% CI | Variance component | 95% CI |
| Within-patient variance | 32.85* | 29.24, 37.53 | 96.55* | 76.75, 124.34* | 267.56* | 189.70, 384.73 |
| Between-patient variance | 20.85* | 15.29, 28.11 | 106.57* | 87.20, 134.47 | 467.88* | 316.21, 683.60 |
| Between-therapist variance | 3.30* | 0.93, 8.89 | 15.57* | 0.96, 39.02 | 26.18* | 2.84, 142.33 |
| ICC | 0.06* | 0.02, 0.15 | 0.08* | 0.01, 0.18 | 0.034* | 0.004, 0.15 |

*Note.* CI = credible interval; ICC = intraclass correlation.

Supplemental Table 2

*Differential Therapist Effects Within the CBT and PDT Conditions*

|  | Clinician-rated Depression | | Patient-rated Depression | | General Psychological Distress | |
| --- | --- | --- | --- | --- | --- | --- |
| **Fixed effects** | Coefficient | 95% CI | Coefficient | 95% CI | Coefficient | 95% CI |
| **CBT condition** |  |  |  |  |  |  |
| Outcome, *γ_000_* | 18.60* | 17.11, 19.80 | 29.98* | 27.02, 33.83 | 69.96* | 59.82, 81.66 |
| Baseline outcome, γ_010_ | 0.82* | 0.49, 1.15 | 0.67* | 0.39, 0.99 | 0.26 | -0.06, 0.56 |
| Medication status, γ_020_ | -2.06 | -5.03, 1.05 | -2.01 | -8.63, 4.05 | 1.35 | -11.41, 14.35 |
| **Random effects** | Variance component | 95% CI | Variance component | 95% CI | Variance component | 95% CI |
| Within-patient variance | 33.22* | 28.91, 38.71 | 100.78* | 82.87, 126.94* | 268.77* | 195.24, 395.84 |
| Between-patient variance | 24.70* | 16.15, 35.53 | 120.66* | 85.33, 190.01 | 548.33* | 283.47, 888.47 |
| Between-therapist variance | 1.04* | 0.13, 4.97 | 13.40* | 1.81, 71.15 | 37.66* | 1.51, 211.70 |
| ICC | 0.02* | 0.002, 0.08 | 0.06* | 0.01, 0.25 | 0.04* | 0.002, 0.22 |
| **PDT condition** |  |  |  |  |  |  |
| Outcome, *γ_000_* | 18.37* | 16.37, 20.22 | 27.94* | 24.76, 30.74 | 68.25* | 59.82, 81.66 |
| Baseline outcome, γ_010_ | 0.85* | 0.50, 1.17 | 0.62* | 0.38, 0.91 | 0.29* | 0.003, 0.52 |
| Medication status, γ_020_ | 1.06 | -2.09, 4.86 | 8.99* | 4.52, 13.88 | 21.51* | 8.43, 35.68 |
| **Random effects** | Variance component | 95% CI | Variance component | 95% CI | Variance component | 95% CI |
| Within-patient variance | 33.22* | 28.91, 38.71 | 100.78* | 82.87, 126.94* | 268.77* | 195.24, 395.84 |
| Between-patient variance | 13.89* | 5.65, 23.57 | 55.08* | 27.29, 92.12 | 341.70* | 174.80, 603.50 |
| Between-therapist variance | 8.75* | 3.37, 20.46 | 28.76* | 4.72, 74.17 | 66.59* | 8.92, 286.54 |
| ICC | 0.16* | 0.06, 0.30 | 0.16* | 0.03, 0.33 | .10* | 0.01, 0.32 |
| Difference in ICCs | 0.14* | 0.03, 0.29 | 0.08 | -0.16, 0.28 | 0.04 | -0.12, 0.26 |

*Note.* CI = credible interval; ICC = intraclass correlation.
